# The World Smarts STEM Challenge: A promising approach to fostering STEM and global competence skills for adolescents in the US and Ghana

**DOI:** 10.1371/journal.pone.0311116

**Published:** 2024-12-02

**Authors:** Esther Kim, Martha Batul, Sarah Bever, Amaris Mohammed, Amanda Nepomunceno, Harrison Owusu, Adam Hartstone-Rose, Kelly Lynn Mulvey

**Affiliations:** 1 North Carolina State University, Raleigh, NC, United States of America; 2 IREX, Washington, DC, United States of America; St John’s University, UNITED STATES OF AMERICA

## Abstract

Given the increasingly global nature of work, the global workforce needs STEM (science, technology, engineering and math) workers who have both STEM content knowledge *and* intercultural competence. This study reports on findings from a 10-week bi-national virtual STEM challenge, the World Smarts STEM Challenge, that brought adolescents in the United States and Ghana together to complete a STEM learning program. There were 114 participants from Ghana (female = 56%) and 95 from the US (female = 48%); *M*_age_ = 16.21 years, *SD* = 1.65. In Ghana 100% of participants identified as ethnically Ghanaian and in the US participants identified as Black/African-American (50%), Latino/a/x or Hispanic (23.9%), Asian/Asian-American (7.6%), White/European-American (7.6%), bi-racial or multi-racial (7.6%), and “other” (3.3%). After the Challenge, participants increased in awareness of global issues, and engagement with others, but also showed a small but significant decrease in respect for people from other cultural backgrounds. Girls demonstrated an increase in global-mindedness in both countries and Ghanaian participants reported an increase in self-efficacy for global issues and demonstrated significant growth in both STEM ability self-concept and STEM activism orientation. Findings suggest the value of virtual STEM Challenges for building both STEM and global competence skills.

## Introduction

Globally, there is a shortage of qualified STEM (science, technology, engineering, and math) workers, with findings suggesting that this shortage is likely to continue [1]. This problem is complex. Certainly, those entering the STEM workforce need concrete STEM skills, but they also need “soft-skills” that enable them to work collaboratively, and on diverse teams [2]. STEM employers consistently highlight gaps both in key STEM competencies as well as teamwork and collaboration skills [3]. Recent latent class analyses indicate that our next generation of STEM workers, adolescents, do not always consistently report both high levels of STEM and interpersonal skills even if they are engaged in STEM programs out of school [4]. Given that the STEM work environment is expected to be increasingly global in coming years [5], to best prepare students for entry into this workforce, efforts must include fostering both STEM skills and global competencies. However, we must also address feelings of capability of stepping into STEM careers: disparities in the representation of women, and ethnic minoritized individuals in STEM majors and in the STEM and information and communication technology (ICT) workforce are pervasive (National Center for Science and Engineering Statistics, 2021). One way to address the need for STEM workers with these skills is by ensuring that adolescents from diverse backgrounds have the interest, skills, and relevant preparation to pursue STEM careers. The current study reports on findings from one effort to address this: a bi-national 10-week virtual STEM experience, the World Smarts STEM Challenge, which partnered adolescents from groups historically excluded from STEM, in the Washington DC Metropolitan area of the United States and in seven regions across Ghana, with a focus on examining outcomes in terms of both STEM and global competencies.

### Contexts: Ghana and the United States

The current study partnered adolescents from two different cultural contexts: in the Washington, DC metropolitan area in the United States and across seven regions (Bono East, Central, Eastern, Greater Accra, Oti, Northern, Savannah, and Volta) in Ghana. While participants came from two countries, within each country there was substantial heterogeneity, with students from different grades as well as different ethnic, religious and economic backgrounds.

In the United States, girls and historically excluded ethnic minoritized students persist in STEM fields at much lower rates than do male and White students [6]. Therefore, the current intervention focused on these populations from the Washington DC area, given that entry into the STEM workforce amongst adolescents from these groups still falls far short of demand for STEM workers, especially in industry and government STEM careers [7]. Further, in Ghana enrollment in higher education is quite limited, especially for girls, who experience few opportunities for STEM advanced education [8–10]. According to UNESCO [11], opportunities for advanced education are strong predictors of key STEM skills, including information and communications technology skills, in low and middle-income countries, including Ghana. ICT skills are lagging in the United States, with findings suggesting up to 1/3 of workers in the US lack key digital skills necessary for their work [12]. Moreover, students in the United States and Ghana do not typically have opportunities to collaborate, partner and learn from and with each other. Therefore, the current study focuses on a bi-national STEM challenge that partnered adolescents in both countries.

### Theoretical frameworks

#### Intergroup contact theory

The World Smarts STEM Challenge was designed to meet the principles of intergroup contact theory [13], which posits that positive intergroup attitudes will develop under certain conditions. Namely, contact theory argues that opportunities for contact must establish equal status and mutual respect between groups, where the groups hold common goals and have opportunities for collaboration, and where the contact is supported by authority figures. Decades of research document that, when the contact conditions are met, positive intergroup attitudes are likely to form [14].

The Challenge was designed for bi-national teams with an equal number of members in the United States and Ghana (equal status) partnered with the support of a mentor in each country (authority sanctioning) to identify a problem aligned with one of the United Nation’s Sustainable Development Goals (common goals) and to work together to develop a prototype solution to address this problem (mutual respect and collaboration). Moreover, on each team, there were equal roles so that one team member in the US and one team member in Ghana each served in parallel roles. For instance, in each country, each team had a “Prototype Developer.” Additionally, all program mentors received training in how to build positive intergroup contact between teams by setting common goals, establishing equal status, encouraging positive interactions and mutual respect, and fostering collaboration. Moreover, both mentors and students also participated in cross cultural communication training, and the curriculum was structured with intentional differentiation between independent, country team, and bi-national team work to ensure equitable participation. The program was designed to engage students in problem-based learning using the human-centered design thinking process and aligned with the principles of intergroup contact theory, with the expectation that meeting the contact conditions would lead to an increase in global competence skills [15] after the intervention. We conceptualized global competence following the PISA Global Competence framework, which frames global competence as involving abilities to understand, and value the viewpoints of others around the world, engagement in positive, appropriate cross-cultural interactions, and both examining and taking action to address global, and intercultural issues, including those focused on sustainability (OECD, 2018). In order to best foster global partnership, the mentor training included training for culturally responsive practices, and the curriculum was assessed by an outside evaluation firm (Catalyst Consulting) as part of a culture audit to ensure that it was culturally sensitive. The culture audit focused on issues such as ensuring materials represent participants, incorporating activities that build group collaboration as well as activities that foster opportunities for student voice and choice.

#### Social cognitive theory

The Challenge is also framed using social cognitive theory [16], which emphasizes the importance of the interaction between people, behavior, and the environmental context in learning. Further, the theory explains that a person’s interest could be shaped by social and environmental factors. Therefore, the Challenge centered on virtual collaboration, creating rich opportunities for interactions between team members as they develop their STEM skills and interest. Following a problem-based learning approach and human-centered design thinking process, the program allowed students to identify and work together to find a solution to a problem in their local communities that aligned with one of the UN’s Sustainable Development Goals. Making STEM relevant to one’s local community and creating opportunities for adolescents to engage in problem-solving locally relevant STEM issues [17, 18], can foster their STEM activism orientation, which refers to their efficacy around being able to address local community problems using STEM solutions and which may help keep them engaged in STEM more broadly [19]. Therefore, the current project created opportunities for STEM behaviors in a locally relevant STEM learning environment, which also fostered opportunities for intercultural partnership and the recognition that STEM problems globally are representative of local STEM problems. Further, the Challenge builds upon social cognitive theory in fostering self-efficacy, namely STEM ability self-concept [20], which has been related to an increased interest in STEM careers over time [21, 22].

### Key program components

#### Virtual cross-cultural exchanges

The Challenge functioned as a hybrid-virtual challenge where adolescents in each country met as a school team or school club in person and joined their binational team members in the partner country virtually. This hybrid approach allowed team members, within-country, to build rapport and collaborate in person, while also drawing on the opportunity for intercultural collaboration with their team members in the other setting. The program aimed to prepare students for a dynamic and changing workforce through virtual project-based learning. Students collaborated and communicated through digital platforms that model the modern global workplace. Research has identified collaborative, virtual learning as an exciting space for education, with findings indicating that online exchange can promote STEM learning [23, 24]. Additionally, following the COVID-19 pandemic, research suggests that adolescents gained considerable skills with virtual learning approaches [25]. Therefore, the Challenge was designed to enable successful virtual cross-cultural exchange.

#### Mentoring in STEM

Another key dimension of the Challenge was a focus on mentoring relationships. Students were paired with a country-based mentor, typically a teacher at their school, who facilitated the virtual exchange in partnership with the mentor in the other country. Additionally, STEM professionals provided supplemental mentoring experiences. For instance, STEM professionals offered a coding workshop for all teams and held a career panel where participants had the opportunity to learn about STEM career possibilities and ask questions of current STEM professionals, for instance by getting assistance on prototype development. Research has indicated the importance of a mentoring relationship [26, 27]. As we live in a society with a shortage of students interested in the STEM field there has been a growing focus on ways to increase STEM interest in adolescents and mentorship has been identified as a highly promising approach [28]. Thus, the Challenge was designed with strong STEM mentorship throughout the program, in order to best foster adolescents’ STEM interest, including STEM activism orientation and self-concept.

### Current study

The aim of the current study was to assess the effects of a STEM out-of-school intervention program in the US and Ghana designed to increase global competence and STEM skills for adolescents. Aligned with research that highlights that not all adolescents, even amongst those who are highly STEM motivated, show high levels of both STEM skills and critical soft-skills needed for interacting in teams and with individuals different from them [4], the current study focused on the interaction between mentors and students and the engagement with international peers. Thus, the study aimed to examine the impact of a cross-cultural team-based STEM program on adolescents’ global competence as well as their STEM interest.

#### Hypotheses

We hypothesized that through the Challenge, participants would 1) increase global competence, including self-efficacy and awareness of global issues, global mindedness, engagement with others regarding global issues, and respect for others from other cultural backgrounds, and 2) demonstrate an increase in STEM orientation, including STEM activism orientation and STEM ability self-concept. Moreover, we also examined if there were differences based on age, country, and gender. We did not expect differences in the global competence measures based on demographics, but we expected that female participants may show more growth than male participants, given that the program may challenge the stereotypes that dissuade women from STEM [29–31]. We did not have specific hypotheses for differences by country or age.

## Materials and methods

### Participants

Students from Ghana and the United States participated in the intervention (*N* = 226). For the analysis, we examined students who completed the whole Challenge (n = 209). There were 114 participants from Ghana (female = 56%) and 95 from the US (female = 48%) between the ages of 11–21 years (*M*_age_ = 16.21 years; Ghana: age range 14 to 21 years, M_age_ = 16.9 and US: age range 11 to 19 years, M_age_ = 15.3). 100% of participants in Ghana self-identified as ethnically Ghanaian and in the US, participants self-identified as Black/African-American (50%), Latino/a/x or Hispanic (23.9%), Asian/Asian-American (7.6%), White/European-American (7.6%), bi-racial or multi-racial (7.6%), and other (3.3%). The sample exceeded a power analysis that indicated that a sample size of at least 88–104 would be necessary for repeated measures ANOVAs with 2 measurements (with correlations from .30-.50) and accounting for gender (2: male, female) and country (2: Ghana, US) with effect sizes at .25 (medium effects) with the desired statistical power at .95, and an alpha of .05 [32]. Data can be accessed at: https://osf.io/8kfes/. This study was approved by the IRB at North Carolina State University (24049). Parents and guardians of participants who were under 18 years of age were provided an opt-out consent form (waiver of signed consent), and participants under 18 years of age electronically assented to participation while participants 18 years of age or older provided electronic consent.

### Design and procedure

After mentors were recruited from both countries, each recruited 8 team members from their local school, with an emphasis on gender-balance and a focus on recruiting students who showed interest in STEM, global connections, virtual exchange, or making a difference in their communities, but who may not have yet had the opportunity to engage in a program like the World Smarts STEM Challenge Before the program began, mentors completed training focused on building capacity to lead the STEM content of the program and cultural competencies around global partnership. Although formal measures of fidelity of implementation were not assessed, site visits were conducted in both countries and mentors received a detailed curriculum guide to follow for each week. Mentors were also paired so that one Ghanaian mentor and one US mentor co-led a team consisting of both US and Ghanaian students. Once students were recruited, they completed a pre-test survey electronically (in Qualtrics) before the program began. The Challenge ran for 10-weeks from January–May 2023. Pre-test data collection began January 6, 2023 and post-test data collection ended on May 31, 2023. Each week, students followed the design thinking process and engaged in series of synchronous and asynchronous activities to understand the needs and opportunities for sustainable solutions in their communities and developed an innovative prototype to address those needs. The curriculum was aligned with the Next Generation Science Standards [33] and explicitly focused on four of them: exploring human impacts on earth systems (ESS3-4), defining and delimiting an engineering problem (ETS1-1), developing possible solutions using engineering design (ETS1-2), and optimizing an engineering design solution (ETS1-3).

The program concluded with two activities, the Virtual Global STEM Exhibit and the Expo. The Virtual Exhibit, which was open to the public through a website, displayed the students’ STEM prototypes through a video pitch and concept paper. During the Virtual Global STEM Expo, the binational teams jointly pitched their STEM prototypes live to a panel of industry professionals and special guests. Following the conclusion of the Challenge, participants completed a post-test survey electronically (also in Qualtrics) using the same measures.

### Measures

#### PISA global competence measure (OECD, 2018)

Global competence was measured with The Program for International Student Assessment (PISA) Global Competence Questionnaire [15], see Table 1. This measure was designed to assess adolescents’ self-efficacy around responding to local, global and intercultural issues, to measure their ability to interact and communicate effectively with people from diverse cultural backgrounds and to take actions to promote global well-being and sustainability. The measure was validated as part of the 2018 PISA assessment with 15-year olds. The subscales of interest for this study assess: 1) self-efficacy regarding global issues, 2) awareness of global issues, 3) global mindedness, 4) engagement with others regarding global issues, and 5) respect for people from other cultural backgrounds. Each subscale has a set of 5- to 8-multi-statement items evaluated on Likert-type scales. Items for each subscale were averaged to create a composite score.

**Table 1:** Measures.

Measure	Example Item	Scoring
PISA Global Competence		
Self-efficacy: global issues	Explain How carbon-dioxide emissions affect global climate change. How easy do you think it would be for you to perform the following tasks on your own?”	1 = I couldn’t do this to 4 = I could do this easily
Awareness of global issues	How informed are you about the following topics? Global warming	1 = I have never heard of this to 4 = I am familiar with this and I would be able to explain this well
Global Mindedness	To what extent do you agree with the following statements? Looking after the global environment is important to me.	1 = Strong disagree to 4 = Strongly agree
Engagement with others regarding global issues	Are you involved in the following activities? I participate in activities in favor of environmental protection.	No = 0, 1 = yes
Respect for people from other cultural backgrounds	How well does each of the following statements below describe you? I treat all people with respect regardless of their cultural background	1 = not at all like me to 5 = very much like me
STEM Activism Orientation	How well do you think your STEM experiences in school have prepared you to do each of the following to solve the problem? Create a plan to address the problem	1 = I definitely can’t to 5 = I definitely can
STEM Ability Self-Concept	How good are you at STEM?	1 = Not at all good to 7 = Very good

The subscale assessing self-efficacy for global issues included six statements (e.g., explain how carbon-dioxide emissions affect global climate change) following the question “How easy do you think it would be for you to perform the following tasks on your own?”. Students’ responses were scored between 1 (“I couldn’t do this”) and 4 (“I could do this easily”; α = .85).

The subscale awareness of global issues included six statements (e.g., climate change and global warming) following the question “How informed are you about the following topics?”. Students’ responses were scored between 1 (“I have never heard of this”) and 4 (“I am familiar with this and I would be able to explain this well”; α = .88).

The subscale assessing global mindedness included six statements (e.g., Looking after the global environment is important to me) following the question “To what extent do you agree with the following statements?”. Students’ responses were scored between 1 (strongly disagree) and 4 (strongly agree; α = .85).

The subscale assessing the participant’s engagement with others regarding global issues included eight statements (e.g., “I participate in activities in favor of environmental protection”) following the question “Are you involved in the following activities?”. Students responded to the statement as “yes” or “no”; α = .80.

Lastly, the subscale assessing respect for people from other cultural backgrounds included five statements (e.g., “I treat all people with respect regardless of their cultural background”) following the question “How well does each of the following statements below describe you?”. Students’ responses were scored between 5 (“very much like me”) and 1 (“not at all like me”; α = .92).

#### STEM activism orientation measure (Mulvey et al., 2022)

For the STEM activism orientation measure, participants read a brief prompt about a community-based STEM problem and then indicated how efficacious they would feel about responding in 8 different ways, see Table 1. First, they read: “If you found out about a problem in your community or school that you wanted to do something about (e.g., high levels of lead were discovered in the local drinking water, or you notice that certain neighborhoods do not have access to a recycling center while others do), how well do you think your STEM experiences in school have prepared you to do each of the following to solve the problem?” Example items include: “Create a plan to address the problem.” and “Apply your STEM knowledge to express your views on the problem.” Students’ responses were scored between 1 (“I definitely can’t”) and 5 (“I definitely can”; α = .92). Scores were averaged to create a composite variable.

#### STEM ability self-concept [20]

Participants completed three items measuring STEM Ability Self-Concept, which were drawn from Eccles and Wang (20) and which have demonstrated validity for use with adolescents, see Table 1. An example item included: “How good at you at STEM?” Responses were scored between 1 (“Not at all good”) to 7 (“Very good”; α = .88). Scores were averaged to create a composite variable.

### Data analytic plan

First, we computed intraclass correlation coefficient (ICC)s and design effects by team to assess if multi-level modeling was needed. Results indicated low levels of variance in outcomes accounted for by team (ICCs ranged from .03 to .10, and all design effects were under 2) which indicated that nesting by team by was not needed. Then, descriptive statistics and correlation analyses were conducted. Following the analyses, we tested our primary hypotheses using 2 (Country: Ghana, US) x 2 (Gender: male, female) X 2 (Time: pre-test, post-test) repeated measures ANOVAs to assess change from pre-to-post intervention and to explore if there were country or gender differences in outcomes. Although age correlated with several of the focal variables (see Table 2), preliminary analyses indicated that age was not a significant covariate in any of the analyses, thus age was dropped from the analyses.

**Table 2:** Descriptive statistics and correlation for study variables.

Variables	1	2	3	4	5	6	7	8	9	10	11	12	13	14
1. Self-efficacy for Global Issues (pre)	-													
2. Awareness of Global Issues (pre)	.61**	-												
3. Global Mindedness (pre)	.42**	.34**	-											
4. Engagement (pre)	.43**	.47**	.42**	-										
5. Respect (pre)	.34**	.23**	.42**	.16**	-									
6. STEM Activism Orientation (pre)	.54**	.49**	.50**	.56**	.23**	-								
7. STEM Ability Self-concept (pre)	.38**	.35**	.43**	.36**	.28**	.46**	-							
8. Self-efficacy for Global Issues (post)	.51**	.50**	.37**	.36**	.31**	.42**	.40**	-						
9. Awareness of Global Issues (post)	.39**	.49**	.30**	.26**	.32**	.32**	.39**	.56**	-					
10. Global Mindedness (post)	.35**	.31**	.43**	.34**	.29**	.31**	.46**	.44**	.44**	-				
11. Engagement (post)	.23**	.25**	.19**	.43**	.04	.25**	.27**	.39**	.14	.41**	-			
12. Respect (post)	.27**	.22**	.28**	.18**	.40**	.18*	.29**	.29**	.31**	.41**	.05	-		
13. STEM Activism Orientation (post)	.37**	.37**	.38**	.25**	.28**	.37**	.52**	.56**	.54**	.57**	.40**	.36**	-	
14. STEM Ability Self-concept (post)	.27**	.22**	.38**	.28**	.25**	.28**	.57**	.42**	.35**	.49**	.38**	.35**	.52**	-

*p < .05.

**p < .01

## Results

### Global competence

In order to examine the effects of the Challenge program on global competence, we conducted a 2 (Country: Ghana, US) x 2 (Gender: male, female) X 2 (Time: pre-test, post-test) ANOVA for the following global competence subscales: 1) self-efficacy regarding global issues, 2) awareness of global issues, 3) global mindedness, 4) engagement with others regarding global issues, and 5) respect for people from other cultural backgrounds (Table 3). For self-efficacy for global issues, results revealed a significant time by country effect, *F* (1, 167) = 6.03, *p* = .015, η_p_^2^ = .04, indicating that Ghanaian participants increased in self-efficacy for global issues from pre-test to post-test, while there were no differences between pre-test and post-test scores for US participants.

**Table 3:** Means for global competence pre- and post-test scores.

	Pre-test Scores			Post-test Scores		
	Ghana *M (SE)*	US *M (SE)*	Total *M (SE)*	Ghana *M (SE)*	US *M (SE)*	Total *M (SE)*
Self-efficacy for Global Issues	3.30 (.057)^a^	3.12 (.062)	3.21 (.042)	3.57 (.051)^a^	3.20 (.055)	3.39 (.037)
Awareness of Global Issues	3.45 (.054)	3.16 (.060)	3.30 (.040)^b^	3.50 (.060)	3.30 (.067)	3.40 (.045)^b^
Global-mindedness	3.54 (.056)	3.18 (.061)	3.36 (.041)	3.64 (.046)	3.20 (.051)	3.42 (.034)
Engagement	1.72 (.029)	1.54 (.032)	1.63 (.022)^c^	1.84 (.025)	1.62 (.028)	1.73 (.019)^c^
Respect	4.87 (.042)	4.70 (.048)	4.78 (.032)^d^	4.80 (.065)	4.47 (.073)	4.63 (.049)^d^

Note: ^a^ Significant difference in self-efficacy for global issues.

^b^ Significant difference in awareness of global issues.

^c^ Significant difference in engagement.

^d^ Significant difference in respect.

Also, there was a significant time effect for the following subscales: 1) awareness of global issues across all participants, *F* (1, 169) = 4.59, *p* = .03, η_p_^2^ = .03, 2) engagement with others, *F* (1, 159) = 19.08, *p* < .001, η_p_^2^ = .11, and 3) respect for people from other cultural backgrounds, *F* (1, 174) = 10.97, *p* = .001, η_p_^2^ = .06, see Table 3. This revealed that participants increased in awareness of global issues, engagement with others from pre-test to post-test. Interestingly, analyses showed a very small, but significant, *decrease* in respect for people from other cultural backgrounds from pre-test to post-test. In addition, results revealed a significant effect of gender for global mindedness, *F* (1, 166) = 3.89, *p* < .05, η_p_^2^ = .02: girls increased in global mindedness from pre-test (*M* = 3.34, *SE* = .056) to post-test (*M* = 3.49, *SE* = .046, *p* = .05), while boys’ scores did not show significant differences from pre-test (*M* = 3.38, *SE* = .061) to post-test (*M* = 3.35, *SE* = .050).

### STEM activism orientation

To test the effects of the Challenge program on STEM activism orientation, we conducted a 2 (Country: Ghana, US) x 2 (Sex: male, female) X 2 (Time: pre-test, post-test) ANOVA (Table 3). Results indicated time by country effect, *F* (1, 166) = 5.67, *p* = .02, η_p_^2^ = .03. This revealed that Ghanaian participants’ scores in STEM activism orientation increased from pre-test to post-test while there were no significant differences between pre-test and post-test scores for US participants.

### STEM ability self-concept

Lastly, we examined the effects of the Challenge program on STEM ability self-concept by conducting a 2 (Country: Ghana, US) x 2 (Sex: male, female) X 2 (Time: pre-test, post-test) ANOVA. Results showed a time by country effect, *F* (1, 175) = 6.94, *p* = .009, η_p_^2^ = .04 (Table 4) with Ghanaian participants reporting higher STEM ability self-concept before the Challenge and also increasing in STEM ability self-concept score from pre-test to post-test while the US participants showed no significant difference in either.

**Table 4:** Means for STEM competence for pre- and post-test.

	Pre-test Scores			Post-test Scores		
	Ghana *M (SE)*	US *M (SE)*	Total *M (SE)*	Ghana *M (SE)*	US *M (SE)*	Total *M (SE)*
STEM Activism Orientation	4.27 (.073)^a^	3.93 (.078)	4.10 (.053)	4.59 (.075)^a^	3.94 (.081)	4.27 (.055)
STEM Ability Self-concept	6.02 (.083)^b^	5.35 (.093)	5.68 (.062)	6.32 (.075)^b^	5.33 (.084)	5.82 (.056)

Note: ^a^ Significant difference in STEM activism orientation

^b^ Significant difference in STEM ability self-concept.

## Discussion

The current study tested outcomes of a novel binational STEM education and cultural virtual program that partnered adolescents in the United States and Ghana over a 10-week period as each team addressed one of UN’s Sustainable Development Goals and built a prototype solution to a challenge they identified aligned with that goal. Our aim was to examine whether participation in this Challenge increased both STEM and global competence skills, and our results affirmed both. Specifically, findings indicated a significant increase in awareness of global issues, and engagement with others, though we found a small but significant decrease in respect for people from other cultural backgrounds from pre-test to post-test for both Ghana and US participants. We also documented that girls demonstrated an increase in global mindedness and that Ghanaian participants reported an increased in self-efficacy for global issues. In terms of STEM competence, we documented that Ghanaian participants especially benefited, demonstrating significant growth from pre-test to post-test in both STEM ability self-concept and STEM activism orientation. While all participants were quite high at baseline on the STEM competence measures, these novel findings are important to highlight because, to our knowledge, no prior research has conducted a cross-cultural virtual learning opportunity for students in Ghana and the US. Moreover, these findings suggest promising results in terms of the potential to increase both the STEM skills widely agreed upon as necessary as well as critical “soft-skills”, namely global competencies, needed to enter the current global STEM workforce.

### Global competence

The Challenge was expected to increase global competence because it gave students the opportunity to work closely with peers from another country, and was designed to meet the intergroup contact theory contact conditions [13]. Specifically, students were paired with mentors who demonstrated authority sanctioning of intergroup contact, had equal status as members of their teams and worked together to collaborate to solve a STEM problem with common goals.

We found that both Ghanaian and US participants showed an increase in awareness of global issues based on a measure that captured how well adolescents thought they have knowledge and understanding of topics such as global health (e.g., epidemics), climate change and global warming. It is important to note that, on average, all participants indicated that they had some knowledge and were aware of global issues before the Challenge (“I know something about this and could explain the general issue”). It may be that participants joined the program with previous knowledge and interest in global issues. However, they did show significant growth in their understanding of global issues. This is likely a result of the direct focus in the curriculum on the UN’s Sustainable Development Goals.

Also, we found that in both countries, participants increased in engagement with others–a set of variables that captured actions one might take to collectively help or engage with protecting the environment and the global population, such as “I participate in activities in favor of environmental protection” and “I regularly read websites on international social issues (e.g., poverty, human rights)”. Aligned with an increase in awareness of global issues, participants were more likely to report that they engage in activities that would increase their knowledge and potential to help address global problems. Thus, students were better equipped to engage in the global world after participating in the Challenges.

Unfortunately, following the Challenge, we found that participants from both countries showed a small, but significant, decrease in respect for people from other cultural backgrounds. The respect for others measure captured how adolescents value other people regardless of their cultural background, such as “I respect the values of people from different cultures.” Importantly, participants overwhelmingly indicated at pre-test that the respect statements were “very much like me” (*M* = 4.79/5, *SD* = .42) and the change by post-test was a very small movement towards “mostly like me” (*M* = 4.65/5, *SD* = .66). Therefore, it is possible that engaging in the challenging work of collaborating cross-culturally allowed participants to recalibrate their expectations slightly to better understand the difficulty of collaboration with individuals from very different cultural backgrounds, a valuable lesson that, despite the slight negative movement, may still serve as preparatory for global engagement. It is also possible that some aspects of the program challenged participants’ perceptions of their respect for other cultures. They may have realized how different their peers in other countries were and experienced some challenges with intercultural interactions that may have slightly reduced their respect for other cultures. Future qualitive research is needed to more thoroughly understand this slight shift. If this slight negative movement demonstrates that some participants prefer not to work cross culturally, this is still a valuable gain in knowledge for those participants. In other words, the participants demonstrated clear successes in solving a STEM problem cross-culturally during the Challenge, and while some participants may be better prepared to join the global STEM workforce, others may have learned the valuable lesson that this career path may not be for them–saving them from the same conclusion that they likely would have learned at a later stage if they did join this field that may be a poor fit for them.

Likewise, we saw unexpected results when examining self-efficacy for global issues, as Ghanaian participants’ scores increased while there were no significant increases in US participants’ scores. This measure captures how capable adolescents thought they would be in doing things such as explaining how carbon-dioxide emissions affect global climate change and discussing the consequences of economic development on the environment. It may be that mentors in Ghana focused more directly on these types of discussions with their team members than did mentors in the United States. It is also possible that US participants felt less prepared on the technical knowledge required to explain the complex processes reflected in this measure. This highlights the importance of more complete fidelity checks in future research to ensure that the curriculum is administered in the same way in both settings. However, it is important to note that while only Ghanaian participants showed significant growth in this measure following the Challenge, both US and Ghanaian participants indicated that they feel that they work somewhat efficaciously with global issues even at baseline (“I could do this with a bit of effort.”).

Additionally, we found that female, but not male, participants showed an increase in global-mindedness—a set of variables that captures desire to help improve the global world, for instance “Looking after the global environment is important to me” and “I think my behavior can impact people in other countries.” Importantly, all participants indicated that they were rather global-minded even before the Challenge (with a mean just above 3 out of 4, “agree”, at pre-test). While female participants showed significant growth in this measure, it may be that male participants take longer to move from learning about global challenges (for instance through the UN’s Sustainable Development Goals) to feeling that they are more invested in taking action to improve these global challenges. Previous literature has shown that girls have higher levels of empathy than do boys [34], which might explain gender differences in global-mindedness in our study.

In sum, findings indicate that the Challenge was effective in increasing both Ghanaian and US adolescents’ skills in thinking about the global world and challenges facing the global world, as well as being ready and interested in engaging with peers from other cultural groups, although more work is needed to understand why participants reported a small decline in respect for other cultures following the challenge.

### STEM competence

Another aim of the current study was to test whether the Challenge increased participants’ STEM competencies. Namely, we measured their STEM Ability-Self Concept [35], which captures how efficacious they feel in STEM domains, generally, as well as their STEM activism orientation, which captures their feelings of competence in being able to serve as local STEM activists and address STEM problems in their community [19]. We focused on a general STEM self-efficacy measure, that may capture more school-related STEM competencies as well as the STEM activism orientation measure, which may capture community-related STEM competencies and included both the STEM ability self-concept and the STEM activism orientation measures given that the Challenge was focused more directly on out-of-school STEM engagement in order to try to capture potential change in both types of STEM competence. We expected that both may be important for the STEM workplace, given that STEM jobs today do often rely on both “academic” STEM skills as well as knowing how to address local STEM problems.

We found that Ghanaian participants showed significant growth in STEM ability self-concept, while US participants’ results did not demonstrate growth. Specifically, this measure captured concepts such as “How good are you at STEM?” Ghanaian participants demonstrated higher levels than US participants at baseline and showed significant growth by post-test, while US participants stayed stable. The increased rates in Ghanaian participants’ results may reflect the fact that Ghanaian participants reported higher levels of STEM orientation and perceiving themselves as more capable, even before the program started. This starting point may have given them higher motivation to continue to develop their STEM ability self-concept during the program. The results may also reflect that US participants showed no growth because they were implicitly comparing themselves to their Ghanaian teammates who were demonstrating higher STEM ability self-concept before the Challenge began.

We found that Ghanaian participants showed a significant increase in STEM activism orientation while US participants’ scores stayed somewhat stable. While both groups started relatively high on this measure indicating that they thought that they might be able to or probably could engage in efforts to address local problems using STEM solutions, Ghanaian students were confident that they “probably” or “definitely could” take action following the challenge. Future research should examine whether there are cultural differences in opportunities and desire to engage in critical actions and, more broadly, in critical consciousness (the ability to recognize and respond to inequality or injustice), between adolescents in the US and Ghana [36, 37]. It may be that feeling capable of engaging in critical actions to address STEM challenges is easier or more culturally appropriate for adolescents in Ghana than in the US. In the US, increasing efforts have been made to give adolescents the opportunity to engage in civic science [17], which may build STEM activism orientation, but this may be because adolescents in the US are especially struggling to feel capable of engaging in civic action to address STEM problems. It may also be that this finding was driven by the STEM Ability self-concept findings; it may be easier to develop STEM activism orientation if you already feel highly efficacious in STEM domains, as the Ghanaian participants did at baseline.

### Limitations

While we documented significant improvement in global and STEM competence by implementing the Challenge, this study had limited fidelity information. For example, it is unclear whether the mentors dedicated the same amount of time to each lesson and implemented them with the same degree of fidelity across teams. Although all mentors completed the same training and reported receiving adequate resources to prepare for the program, several mentors requested more information post-training and provided feedback about needing more time for implementation [38]. Therefore, it may be that not all teams experienced the curriculum in the same way.

Also, we included a wide age range of adolescents in the study and while our study did not show any significant age differences in global and STEM competence, it is possible that younger adolescents might have had different experiences compared to older adolescents throughout the program. In addition, Ghanaian participants were older (age range 14 to 21, *M*_age_ = 16.9) than were the US participants (age range 11 to 19, *M*_age_ = 15.3), in alignment with the typical ages that students are enrolled in secondary school in both countries. Therefore, future research should aim to test a balanced age range of adolescents across countries and investigate differences in STEM curricula across schools and countries.

One strength of our study is that the Challenge was conducted between students from Ghana and the United States entirely virtually. While participants were given the opportunity to engage with others from a different country, the bi-national component of the program was entirely virtual and students may have experienced fatigue from virtual learning. This may especially be the case for the US where schools were entirely virtual for up to two years due to COVID-19. A majority of schools and homes in Ghana have limited to no internet access. Therefore, other platforms such as the radio and television were also used to deliver lessons during the pandemic [39]. For example, The Ghana Education Service (GES) with support from the government created a TV channel to broadcast free educational content throughout the day. In addition, private online platforms and radio broadcasting also helped deliver educational content. Therefore, Ghanaian students who did not have access to the internet received lessons through different platforms, which may have decreased potential burnout relative to US students who were subjected predominantly to one type of virtual learning medium.

### Implications and conclusions

The findings suggest that hybrid international virtual education and exchange programs incorporating design thinking and solving real world problems can be effective at building STEM and global competency skills for adolescents. Over a 10-week period in the World Smarts STEM Challenge, students from both the US and Ghana investigated, collaborated, interacted, and designed a STEM prototype solution to a real and SDG related problem they identified in their community. The Challenge provided rich opportunities to collaborate and connect with peers in a very different setting than their usual educational experiences and to develop key STEM and cross-cultural skills. It is clear that we need workers prepared with both STEM skill sets and skills sets that facilitate interaction and engagement with people who are different than oneself in the STEM workforce. The World Smarts STEM Challenge is a promising new model for this kind of global STEM learning.

Moreover, the findings suggest the potential value of integration of global competence skills into traditional STEM curricula, in order to better prepare youth to enter STEM careers. While not all teachers will be able to launch a bi-national challenge such as the World Smarts STEM Challenge, they can look to create opportunities for intergroup contact for their students, expose them to international STEM professionals for instance through “Zoom a Scientist” sessions, and integrate the Sustainable Development Goals into their lessons to highlight how the world’s most pressing STEM challenges are both locally and globally relevant. Additionally, the findings highlight the importance of providing STEM interventions to youth who have the potential to be tomorrow’s STEM workers, but who may not yet have realized their STEM interests. While the vast majority of participants in the World Smarts STEM Challenge expressed high STEM and global competence skills at pre-test and increased these skills across the program, there are likely many students who would benefit even more and who have unrealized potential as future STEM workers. Finally, the program suggests that interdisciplinary curricula that foster both STEM and interpersonal skills together can be engaging, exciting and beneficial ways to promote STEM and global competence for today’s students across the globe, including women and students from historically excluded backgrounds.
